# Macroecological synchronization of vector-borne diseases under climate variability in Colombia

**DOI:** 10.1016/j.soh.2026.100169

**Published:** 2026-09-05

**Authors:** Mario J. Olivera, Julián Felipe Porras-Villamil, Màrius Vicent Fuentes

**Affiliations:** a Grupo de Parasitología, Instituto Nacional de Salud, Bogotá 111321, Colombia; b Parasites and Health Research Group, Facultat de Farmàcia i Ciències de l'Alimentació, Universitat de València, València 46010, Spain; c Department of Entomology, University of Kentucky, Lexington, KY 40546, USA

**Keywords:** Climate change, El Niño–Southern oscillation, Malaria, Cutaneous leishmaniasis, Chagas disease, Colombia

## Abstract

**Background:**

Climate variability may synchronize vector-borne diseases across distinct transmission systems. This study evaluates climatic coherence among malaria, cutaneous leishmaniasis, and Chagas disease in Colombia and projects changes in environmental suitability under future climate scenarios.

**Methods:**

National surveillance, climatic, environmental, and socioeconomic data from 1980 to 2025 were analyzed using generalized additive models, distributed lag nonlinear models, and wavelet coherence. Annual series were used for interannual analyses, whereas monthly epidemiological and climatic series were used to evaluate delayed associations. Ecological niche models for selected vector species were projected to 2035 under three Coupled Model Intercomparison Project Phase 6 (CMIP6) climate scenarios.

**Results:**

El Niño–Southern Oscillation (ENSO) was associated with temperature, precipitation, and relative humidity anomalies. Incorporating ENSO variability improved model performance for malaria and cutaneous leishmaniasis beyond models based only on local climatic predictors. Delayed climatic responses differed among diseases; the most robust association was observed for malaria, with a 1 °C increase in temperature anomaly associated with 28.3% higher incidence over 3–8 months (relative risk [RR] = 1.28; 95% confidence interval [*CI*]: 1.03–1.60). Malaria showed the strongest temporal coherence with ENSO, followed by cutaneous leishmaniasis, whereas Chagas disease showed weaker patterns. By 2035, high-suitability areas increased by 6.2–9.8% for malaria and 8.8–16.8% for cutaneous leishmaniasis but decreased by 14.7–24.9% for Chagas disease. Spatial overlap remained high (mean Schoener's *D* = 0.886 under each scenario), while projected multi-vector co-occurrence ranged from 36,185 to 52,142 km^2^ across the three 2035 scenarios.

**Conclusion:**

ENSO was associated with macroecological synchronization across transmission systems, although climatic responses differed among diseases. Future projections indicate spatial reorganization rather than uniform expansion, supporting integrated, climate-informed surveillance in areas of overlapping environmental suitability.

## Graphical abstract

**Figure:**
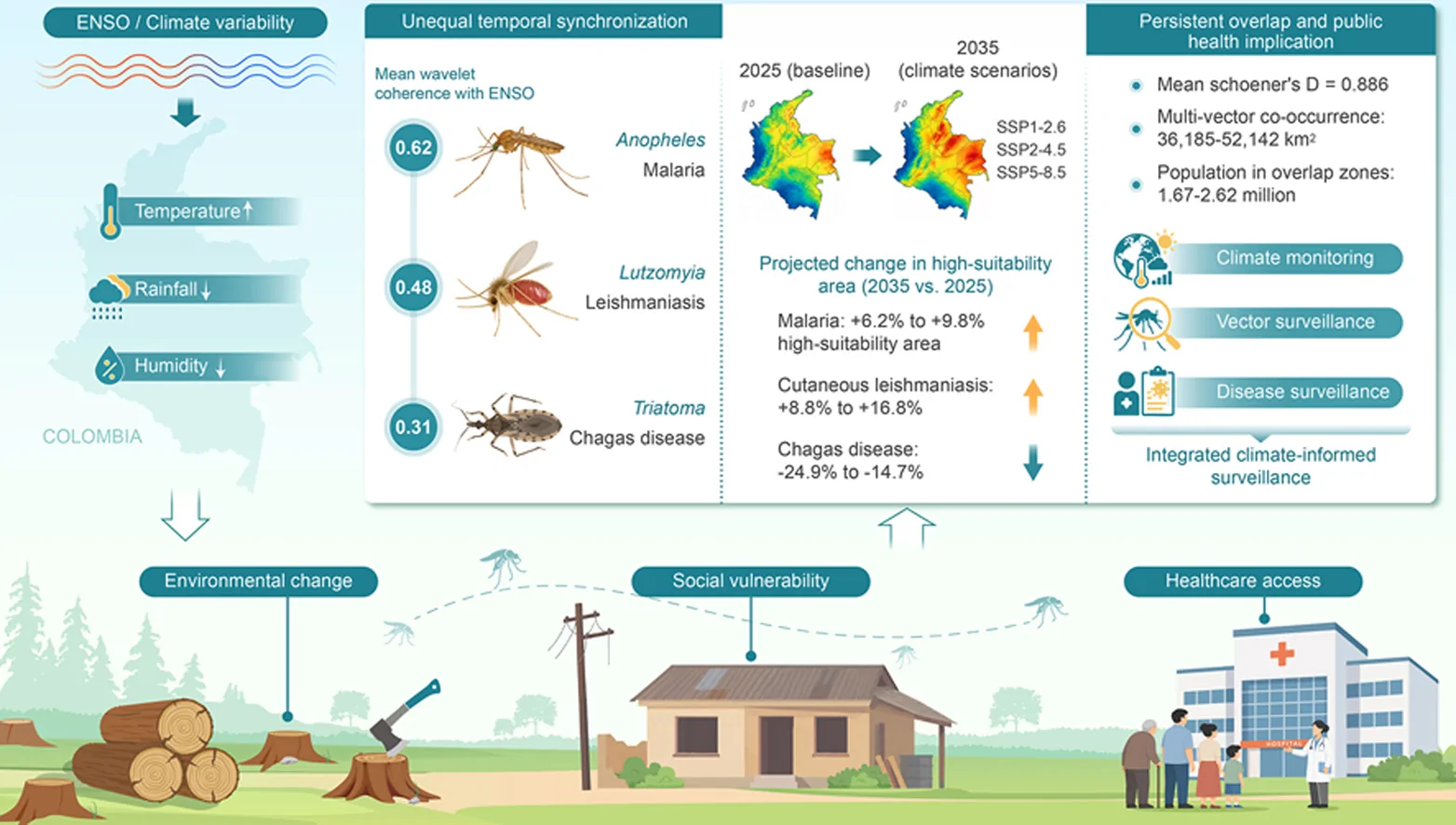

## 1 Introduction

Climate variability is reshaping the global geography of vector-borne diseases by altering temperature, rainfall, and ecosystem stability in ways that increasingly link distant transmission systems under shared environmental forcing [[1], [2], [3]]. These environmental shifts affect vector survival, parasite development, and human exposure, reshaping spatial and temporal transmission patterns [4,5]. More than 80% of the global population resides in areas exposed to at least one vector-borne pathogen, underscoring the climatic influence on public health [6].

In tropical Latin America, the El Niño–Southern Oscillation (ENSO) modulates rainfall and temperature, influencing the ecology of mosquitoes, sandflies, and triatomines [[7], [8], [9], [10]]. These disturbances alter breeding environments and humidity, producing recurrent interannual incidence fluctuations [11,12]. While many studies describe climatic sensitivity of individual diseases [13,14], it remains unclear whether large-scale climatic variability generates coordinated responses across multiple transmission systems with distinct ecological dynamics [5,10].

Colombia encompasses Pacific, Andean, and Amazon ecoregions and sustains concurrent endemic transmission of malaria (*Plasmodium* spp.), cutaneous leishmaniasis (*Leishmania* spp.), and Chagas disease (*Trypanosoma cruzi*) [[15], [16], [17]]. The combination of steep altitudinal and climatic gradients exposes distinct parasite–vector systems to overlapping environmental constraints [18,19]. This convergence of ecological gradients and co-endemic diseases provides a natural setting to examine how large-scale climatic variability structures multi-disease dynamics across heterogeneous transmission systems [20,21].

Distinct regional profiles are evident. Malaria predominates in the Pacific and Amazon, associated with riverine settlements and extractive economies [16]. Cutaneous leishmaniasis clusters along agricultural and deforestation frontiers, affecting dispersed rural communities in forested settings [17]. Chagas disease persists in Andean and Orinoco foothills, where vector-borne transmission continues despite substantial reductions in intradomiciliary infestation by *Rhodnius prolixus* [15,22,23]. Despite these differences, their overlapping rural distributions suggest potential shared responses to environmental variability, highlighting the need for integrated, multi-disease surveillance approaches.

These interactions highlight the coupling of human and environmental systems, yet operational frameworks integrating climatic and ecological information into disease surveillance remain limited [24]. Moreover, current approaches are largely disease-specific, limiting the ability to detect coordinated responses driven by shared climatic forcing. This study introduces the concept of macroecological synchronization, referring to correlated spatial and temporal variation among distinct disease systems associated with large-scale climatic oscillations such as ENSO [10].

Building on this framework, this study aimed to determine whether ENSO-related climatic variability acts as a unifying environmental driver shaping the spatial and temporal dynamics of multiple vector-borne diseases simultaneously. The study evaluates long-term epidemiological patterns and their association with climatic oscillations and assesses the potential for environmental change to reshape the geographic overlap of transmission systems. By integrating multiple diseases within a common analytical framework, the study advances understanding of climate–disease interactions beyond single-disease perspectives and provides a basis for climate-informed, multi-system surveillance strategies in tropical regions.

## 2 Methods

### 2.1 Study design and framework

A comparative ecological design was used, integrating epidemiological, climatic, environmental, entomological, and socioeconomic data from 1980 to 2025. The analytical framework combined temporal and spatial analyses through three complementary components: (i) national-scale temporal modeling of disease incidence, (ii) spatial habitat suitability modeling for vector species, and (iii) municipality-level socioecological assessment.

The framework was structured to evaluate a hierarchical pathway linking large-scale climatic variability (ENSO), local climatic anomalies (temperature, precipitation, and relative humidity), and disease incidence, while assessing temporal and spatial synchronization across disease systems. This approach allowed the relative contributions of large-scale climatic forcing, local environmental conditions, and disease-specific dynamics to be evaluated across complementary analytical scales.

### 2.2 Data

Monthly case counts for malaria, cutaneous leishmaniasis, and Chagas disease were obtained from the Parasitology Group of the National Institute of Health for 1980–2006 and from the National Public Health Surveillance System for 2007–2025. The complete monthly series comprised 552 observations (January 1980–December 2025) for each disease. Disease-specific annual populations at risk were derived from population projections from the National Administrative Department of Statistics and were used to calculate annual incidence rates (cases per 100,000 population at risk).

Georeferenced vector occurrence records were compiled from the Global Biodiversity Information Facility and the Entomology Group of the National Institute of Health. Environmental predictors used for ecological niche modeling included mean annual temperature (BIO1), temperature seasonality (BIO4), precipitation of the wettest quarter (BIO16), relative humidity, vegetation, deforestation, and elevation. Climatic predictors were obtained from WorldClim v2.1 and CHELSA v2 at 1 km^2^ spatial resolution and bias-corrected using meteorological observations from the Institute of Hydrology, Meteorology, and Environmental Studies. Vegetation indices and deforestation data were derived from the Moderate Resolution Imaging Spectroradiometer (MODIS) and Global Forest Watch, respectively, and elevation data from the Shuttle Radar Topography Mission.

Temperature, precipitation, and relative humidity were available as monthly climatic series. Monthly anomalies were derived from the corresponding series and aggregated by calendar year when required for annual temporal analyses. Monthly Oceanic Niño Index (ONI) values were obtained from the National Oceanic and Atmospheric Administration and aggregated to annual values for analyses of interannual ENSO variability.

Socioeconomic indicators included poverty, population density, and health access at the municipality level. Data were obtained from national statistical and administrative sources.

All raster layers were resampled to 1 km^2^ resolution and reprojected to the World Geodetic System 1984 (WGS 84). Climatic, environmental, and socioeconomic predictors included in the modeling framework are described in Supplementary Table S1. For reference, the geographic distribution of Colombia by administrative departments and natural regions is shown in Supplementary Fig. S1.

### 2.3 ENSO–climate relationships

Associations between annual ONI anomalies and national-scale anomalies of temperature, precipitation, and relative humidity were quantified. Pearson correlation analyses and generalized additive models (GAMs) were used to evaluate these relationships and capture potential nonlinear responses. Temperature, precipitation, and relative humidity anomalies were analyzed as dependent variables, with ONI anomalies specified as the principal explanatory variable. GAMs were fitted using penalized regression splines, with smoothing parameters estimated through restricted maximum likelihood to characterize potentially nonlinear relationships between ENSO variability and local climatic anomalies [25]. These analyses quantified the extent to which large-scale ENSO variability was associated with local climatic anomalies within the proposed hierarchical framework linking macroclimatic variability and local environmental conditions. Statistical significance was assessed at α = 0.05.

### 2.4 National temporal modeling of disease incidence

National temporal dynamics were evaluated at complementary annual and monthly resolutions. Annual incidence rates were log-transformed [log (I_t_ + 1)] to stabilize variance. Temporal trends were decomposed into long-term, interannual, and residual components using locally weighted regression, and structural changes in disease trajectories were identified using segmented regression models. Breakpoints were estimated iteratively, and model selection was based on Bayesian information criterion minimization [26]. Uncertainty in breakpoint location was quantified through nonparametric bootstrap resampling (1000 iterations), generating empirical 95% confidence intervals (*CI*s).

Annual incidence series were used to evaluate associations with interannual climatic variability. For each disease, two GAMs were fitted to evaluate local climatic associations and the additional contribution of large-scale ENSO variability. Both models included the same penalized smooth of calendar year to account for nonlinear long-term temporal trends. Model 1 included annual temperature, precipitation, and relative humidity anomalies as parametric predictors, whereas Model 2 retained the same temporal smooth and local climatic predictors and additionally incorporated annual ONI anomalies. Model performance was compared using the Akaike information criterion (AIC), with negative ΔAIC values indicating improved fit of Model 2 relative to Model 1. Residual diagnostics indicated approximately Gaussian residual distributions following transformation. The models were specified as follows:

$$\;\text{Model}\;1:\log\left(I_{t}+1\right)=\beta_{0}+s\left(Year_{t}\right)+\beta_{1}Temp_{t}+\beta_{2}Prec_{t}+\beta_{3}RH_{t}+\varepsilon_{t}$$

$$\;\text{Model}\;2:\log\left(I_{t}+1\right)=\beta_{0}+s\left(Year_{t}\right)+\beta_{1}Temp_{t}+\beta_{2}Prec_{t}+\beta_{3}RH_{t}+\beta_{4}ONI_{t}+\varepsilon_{t}$$

where $I_{t}$ denotes annual disease incidence in year t; $s\left(Year_{t}\right)$ represents the penalized smooth function of calendar year; $Temp_{t}$, $Prec_{t}$, and $RH_{t}$ denote annual temperature, precipitation, and relative humidity anomalies, respectively; $ONI_{t}$ denotes the annual Oceanic Niño Index anomaly; and $\varepsilon_{t}$ represents the error term

Delayed associations between local climatic variability and disease incidence were evaluated separately at monthly resolution using distributed lag nonlinear models (DLNMs) [27]. Monthly disease counts were modeled using single-exposure quasi-Poisson models, with the corresponding disease-specific annual population at risk included as a log offset. Temperature, precipitation, and relative humidity anomalies were evaluated separately using cross-basis functions with natural cubic splines for the exposure–response and lag–response dimensions (3 df each). A maximum lag of 12 months was specified. The complete monthly series comprised 552 observations (January 1980–December 2025); consequently, the first 12 observations did not have complete lag histories and were excluded from model estimation, yielding 540 observations per disease model. Models controlled for long-term temporal trends using a natural cubic spline with 1 df per year and for seasonality using annual and semiannual harmonic terms.

Effect estimates were expressed as cumulative relative risks (RRs) for a 1 °C increase in temperature anomaly, a 10 mm increase in precipitation anomaly, and a 1-percentage-point increase in relative humidity anomaly. Cumulative associations were estimated across the complete 0–12-month lag horizon. Associations over lags 3–8 months were additionally estimated to characterize delayed responses within this interval. Because residual serial dependence remained in the monthly models, 95% *CI*s were calculated using Newey–West heteroskedasticity- and autocorrelation-consistent (HAC) covariance estimators with a 12-month lag. Model robustness was evaluated through negative binomial sensitivity analyses as described below.

### 2.5 ENSO–disease associations

Effect estimates for annual ENSO–disease associations were derived from Model 2 and expressed as percentage changes in incidence associated with a one-unit increase in ONI. Percentage changes were calculated from exponentiated model coefficients and are reported with 95% *CI*s and corresponding changes in AIC (ΔAIC).

### 2.6 Temporal synchrony and periodicity analysis

Wavelet power spectra and wavelet coherence analyses were conducted to evaluate synchronization between ENSO variability, climatic variables, and disease incidence.

Prior to wavelet analyses, epidemiological and climatic time series were standardized as z-scores [z_t_ = (y_t_ − mean(y))/SD(y)] to facilitate comparison among variables with different magnitudes and variances and to ensure consistency with coherence estimation procedures. For visualization purposes, the annual epidemiological and climatic series displayed in Fig. 1 were standardized using the same transformation.

**Fig. 1:**
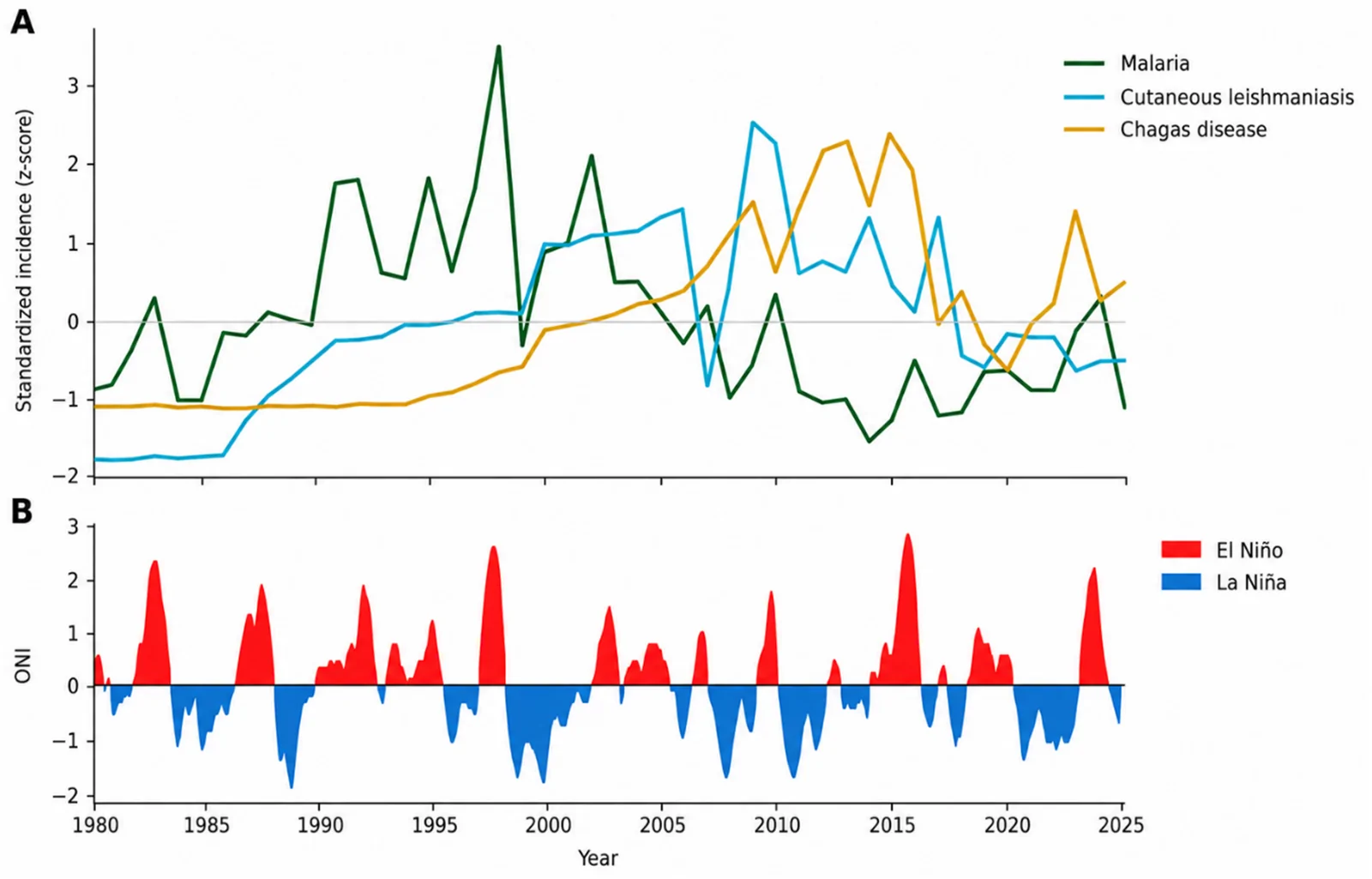
**Temporal dynamics of malaria, cutaneous leishmaniasis, and Chagas disease incidence and El Niño–Southern Oscillation (ENSO) variability in Colombia, 1980–2025** (A) Standardized annual incidence trajectories (z-scores) of malaria, cutaneous leishmaniasis, and Chagas disease (B) Oceanic Niño Index (ONI) anomalies showing El Niño (positive values) and La Niña (negative values) phases.

Wavelet coherence was used to identify shared periodicities and non-stationary coupling across time-frequency space. Analyses focused on periodicities between 2 and 8 years, corresponding to the dominant temporal scales associated with ENSO variability. Statistical significance was assessed using Monte Carlo permutation tests (1000 iterations).

Continuous wavelet transforms and coherence estimation procedures commonly applied to non-stationary environmental time series were used to characterize temporal synchronization patterns [28].

### 2.7 Ecological niche modeling and future climatic suitability

Ecological niche models were developed for the principal vector species associated with malaria, cutaneous leishmaniasis, and Chagas disease in Colombia using the maximum entropy (MaxEnt) algorithm [29]. Occurrence records were obtained from national surveillance databases and the Global Biodiversity Information Facility. Environmental predictors included mean annual temperature (BIO1), temperature seasonality (BIO4), precipitation of the wettest quarter (BIO16), relative humidity, Normalized Difference Vegetation Index (NDVI), deforestation, and elevation. Collinearity among predictors was assessed (|r| ≥ 0.75), and variables with stronger biological interpretability were retained.

Model performance was evaluated using the area under the receiver operating characteristic curve (AUC >0.85) and the true skill statistic (TSS >0.70), with five-fold cross-validation.

Projections for 2035 were generated under three Coupled Model Intercomparison Project Phase 6 (CMIP6) Shared Socioeconomic Pathway (SSP) scenarios (SSP1–2.6, SSP2–4.5, and SSP5–8.5) using MPI-ESM1-2-HR, the CMIP6 source identifier for the higher-resolution version of the Max Planck Institute Earth System Model version 1.2 (MPI-ESM1.2-HR). Projections were interpreted as relative environmental suitability rather than direct estimates of disease burden.

Environmental suitability projections were generated independently for each vector species and rescaled to a common 0–1 range. Species-specific projections were subsequently combined into disease-specific ensemble suitability surfaces. Areas with ensemble suitability values ≥ 0.70 were classified as high-suitability areas, and changes in their extent between 2025 and 2035 were quantified for each disease and climate scenario. Suitability-weighted mean elevation was calculated from the continuous disease-specific suitability surfaces for 2025 and 2035, and the difference between these values was used to characterize projected elevational shifts.

Spatial convergence among disease-specific suitability surfaces was quantified using pairwise Schoener's *D* calculated from the normalized continuous suitability surfaces, with the mean of the three pairwise values used as an overall overlap measure for each scenario. Multi-vector co-occurrence was evaluated separately using threshold-based suitability classification, and the corresponding co-occurrence area and population residing within these zones were quantified for each climate scenario.

### 2.8 Municipality-level socioecological modeling

A pooled municipality-level negative binomial generalized linear mixed-effects model was fitted across the three disease systems. Disease system was included as a fixed effect to account for baseline differences among malaria, cutaneous leishmaniasis, and Chagas disease. Main effects for temperature anomaly, precipitation anomaly, deforestation, poverty, and health access represented associations across disease systems. Prespecified interaction terms between climatic anomalies and environmental or socioeconomic conditions were included to assess effect modification, with disease-specific interaction estimates reported where relevant. Municipality was included as a random intercept, and the disease-specific municipality-year population at risk was incorporated as a log offset. All disease counts and predictors were organized at annual temporal resolution.

$$\log\left[E\left(Y_{idt}\right)\right]=\log\left(Pop_{idt}\right)+\beta_{0}+\gamma_{d}Disease_{d}+\beta_{1}Temp_{it}+\beta_{2}Prec_{it}+\beta_{3}Defor_{it}+\beta_{4}Poverty_{it}+\beta_{5}Health_{it}+\sum_{k}\delta_{k}Interaction_{kidt}+u_{i}$$

where $Y_{idt}$ denotes the annual number of cases for disease system d in municipality i and year t; $Pop_{idt}$ is the corresponding disease-specific population at risk; $Disease_{d}$ represents the fixed effect for disease system; $Temp_{it}$, $Prec_{it}$, $Defor_{it}$, $Poverty_{it}$, and $Health_{it}$ denote the municipality-year predictors; $Z_{kidt}$ represents the prespecified interaction terms; and $u_{i}$ is the municipality-specific random intercept.

### 2.9 Quantification of macroecological synchronization

To operationalize the concept of macroecological synchronization, convergence was evaluated across complementary temporal and spatial dimensions. Temporal synchronization was assessed through wavelet coherence within the prespecified 2–8-year periodicity range, including differences in dominant periodicity, coherence magnitude, and temporal persistence among disease systems. Spatial convergence was quantified using overlap metrics derived from the disease-specific ecological suitability surfaces described above. These indicators were used to compare temporal synchronization and spatial convergence patterns among diseases.

### 2.10 Robustness analyses

For spatial visualization, incidence rates were transformed into disease-specific percentile ranks ranging from 0 to 100, allowing comparison of relative spatial incidence patterns across diseases without implying equivalence in absolute incidence levels.

For monthly DLNMs, robustness was evaluated using negative binomial models with the same exposure, lag, temporal, seasonal, and population-offset specification as the primary quasi-Poisson models.

Given the hypothesis-driven nature of the analyses and the limited number of predefined comparisons, no correction for multiple testing was applied.

### 2.11 Software

All analyses were conducted using R version 4.3.2 (R Foundation for Statistical Computing, Vienna, Austria) and QGIS version 3.34 (QGIS.ORG, Laax, Switzerland). Generalized additive models were implemented using mgcv, distributed lag nonlinear models using dlnm, HAC covariance estimation using sandwich, negative binomial sensitivity models using MASS, segmented regression using segmented, wavelet coherence analyses using WaveletComp, ecological niche modeling using maxnet and ENMeval, and generalized linear mixed-effects models using glmmTMB.

### 2.12 Ethics statement

This study used exclusively anonymized secondary surveillance and environmental data and involved no direct interaction with human participants. Under Resolution 8430 of 1993 of the Colombian Ministry of Health, the study was classified as research without risk. No individual-level identifiable information was accessed, and informed consent was not applicable to these secondary-data analyses [30].

## 3 Results

### 3.1 ENSO–climate relationships (1980–2025)

Long-term epidemiological and climatic patterns showed clear ENSO-related structuring across Colombia. Between 1980 and 2025, Colombia reported approximately 4.7 million malaria cases, 313,000 cases of cutaneous leishmaniasis, and 16,173 cases of Chagas disease. Malaria exhibited the highest incidence and temporal variability, followed by cutaneous leishmaniasis, whereas Chagas disease showed lower incidence but greater relative variability over time (Supplementary Table S2).

Annual ONI anomalies were significantly associated with national-scale temperature, precipitation, and humidity anomalies (Table 1). A one-unit increase in ONI was associated with a mean increase of 0.38 °C in temperature anomalies (95% *CI*: 0.30–0.45; *P* < 0.001), a decrease of 42 mm in precipitation (95% *CI*: −55 to −28; *P* < 0.001), and a 1.8-percentage-point decrease in relative humidity anomalies (95% *CI*: −3.1 to −0.6; *P* = 0.004). ENSO-related variability was consistently expressed in temperature and precipitation anomalies, with comparatively weaker contributions from humidity.

**Table 1:** Association between ENSO variability (ONI) and local climatic anomalies in Colombia, 1980–2025.

Predictor	Effect estimate	95% *CI*	*P*-value
Temperature anomaly (°C per +1 ONI)	0.38	0.30–0.45	<0.001
Precipitation anomaly (mm per +1 ONI)	−42	−55 to −28	<0.001
Relative humidity anomaly (percentage points per +1 ONI)	−1.8	−3.1 to −0.6	0.004

**Note:** Effect estimates represent the mean change in each climatic variable associated with a one-unit increase in annual ONI anomaly.

**Abbreviations:** CI, confidence interval; ENSO, El Niño–Southern Oscillation; ONI, Oceanic Niño Index.

### 3.2 ENSO–disease relationships

#### 3.2.1 Temporal incidence patterns

Temporal incidence trajectories showed distinct disease-specific responses throughout the study period. Malaria incidence exhibited pronounced interannual variability, with major peaks coinciding with strong El Niño episodes, particularly in 1983 and 1997–1998, and secondary increases in 2010 and 2024 (Fig. 1). Cutaneous leishmaniasis displayed recurrent interannual fluctuations throughout the study period, whereas Chagas disease showed comparatively lower incidence but a gradual increase after 2000.

Temporal correspondence between major ENSO events and periods of increased malaria and cutaneous leishmaniasis incidence was evident, whereas associations appeared weaker for Chagas disease.

#### 3.2.2 Temporal incidence models

Results from Model 1, which included only local climatic anomalies (temperature, precipitation, and relative humidity), are presented in Supplementary Table S3. Temperature anomalies were positively associated with malaria and cutaneous leishmaniasis incidence, whereas precipitation anomalies showed predominantly negative associations. Associations with Chagas disease were weaker and not statistically significant. Model 1 explained 57.3% of the deviance in malaria incidence, 55.7% in cutaneous leishmaniasis, and 45.6% in Chagas disease.

Results from Model 2, which additionally incorporated annual ONI anomalies beyond local climatic variables (Table 2), indicated that a one-unit increase in ONI was associated with a 6.1% increase in malaria incidence (95% *CI*: 2.3–9.8; *P* = 0.002) and a 4.3% increase in cutaneous leishmaniasis incidence (95% *CI*: 1.1–7.5; *P* = 0.009), whereas no statistically significant association was observed for Chagas disease (−2.0%; 95% *CI*: −4.8 to 0.7; *P* = 0.140). Model fit improved after incorporation of ONI anomalies, with ΔAIC values of −12 for malaria, −8 for cutaneous leishmaniasis, and −2 for Chagas disease, indicating that ENSO variability contributed additional explanatory power beyond local climatic predictors.

**Table 2:** Generalized additive model estimates of the association between ENSO variability and annual disease incidence in Colombia, 1980–2025.

Disease	ONI effect (% change per +1 ONI)	95% *CI*	*P*-value	ΔAIC vs. Model 1
Malaria	6.1	2.3–9.8	0.002	−12
Cutaneous leishmaniasis	4.3	1.1–7.5	0.009	−8
Chagas disease	−2.0	−4.8 to 0.7	0.140	−2

**Note:** Estimates represent the percentage change in annual disease incidence associated with a one-unit increase in annual ONI anomaly in Model 2, adjusted for local temperature, precipitation, and relative humidity anomalies. ΔAIC values compare Model 2 with the corresponding disease-incidence Model 1, which included the same local climatic predictors but did not include ONI; negative ΔAIC values indicate improved model fit after inclusion of ONI.

**Abbreviations:** AIC, Akaike information criterion; *CI*, confidence interval; ENSO, El Niño–Southern Oscillation; ONI, Oceanic Niño Index.

Monthly DLNMs provided complementary evidence of disease-specific delayed associations with local climatic variability (Supplementary Table S4). Across the complete 0–12-month lag horizon, none of the cumulative associations remained statistically significant using HAC CIs. Within lags 3–8 months, a 1 °C increase in temperature anomaly was associated with a 28.3% increase in malaria incidence (RR = 1.28; HAC 95% *CI*: 1.03–1.60). Higher relative humidity was associated with lower cutaneous leishmaniasis incidence over the same lag window (RR per 1-percentage-point increase = 0.89; HAC 95% *CI*: 0.78–1.00), although the estimate was borderline under autocorrelation-robust inference. No statistically significant delayed associations were identified for Chagas disease over lags 3–8 months. Negative binomial sensitivity models yielded comparable estimates, particularly for the malaria–temperature association (RR = 1.30; 95% *CI*: 1.14–1.49).

#### 3.2.3 Wavelet-based temporal synchronization

Wavelet coherence analyses provided complementary evidence of temporal synchronization between ENSO variability and disease incidence across interannual periodicities.

Pairwise coherence analyses identified statistically significant coupling between ONI variability and malaria incidence within the 3–5-year periodicity band, consistent with characteristic ENSO temporal scales. Coherence persisted across multiple temporal intervals throughout the study period (Fig. 2A).

**Fig. 2:**
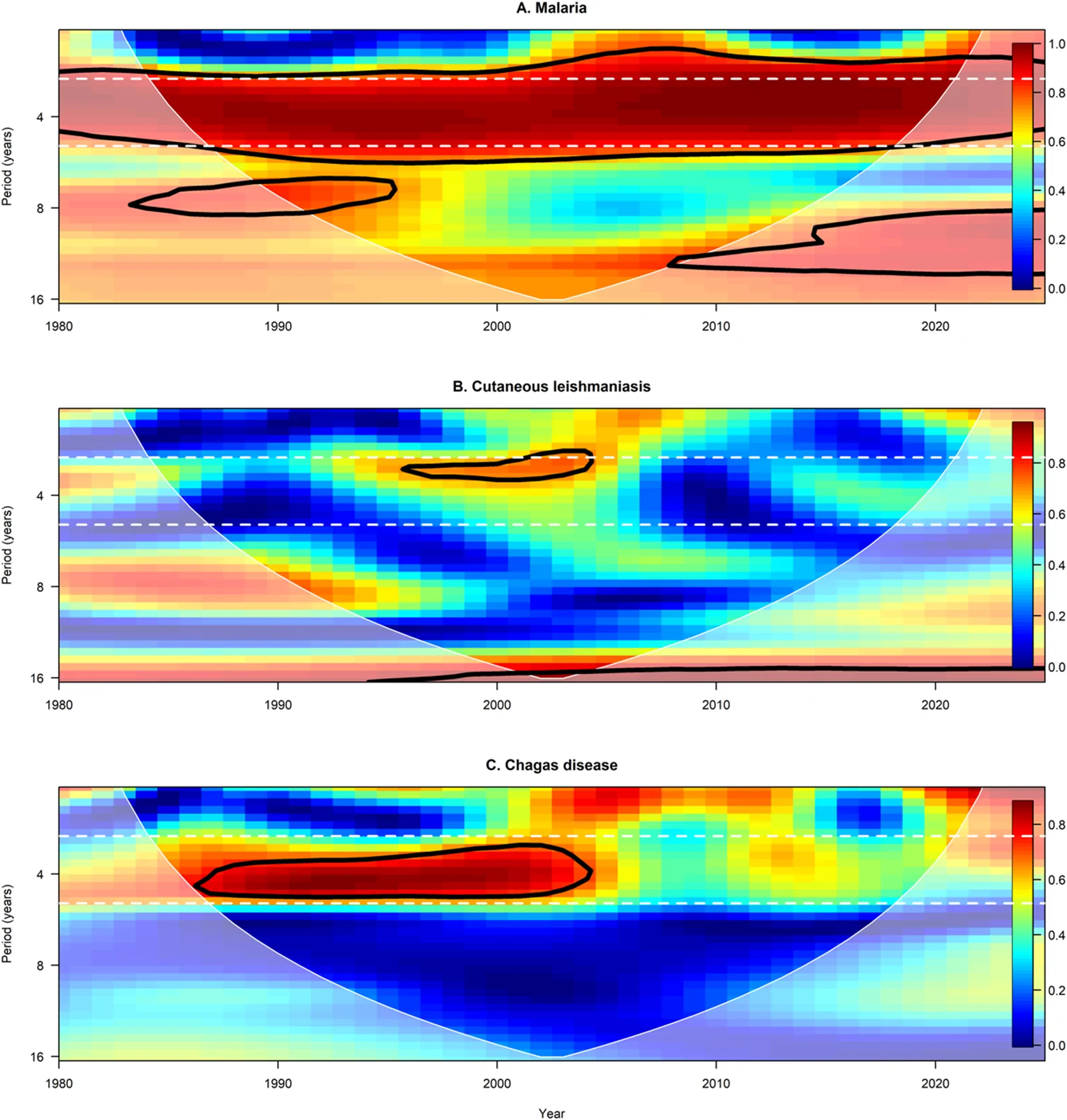
**Wavelet coherence between El Niño–Southern Oscillation (ENSO) variability and incidence of malaria, cutaneous leishmaniasis, and Chagas disease in Colombia, 1980–2025.** Coherence was estimated between Oceanic Niño Index (ONI) anomalies and incidence of (A) malaria (B) cutaneous leishmaniasis, and (C) Chagas disease. Warm colors indicate higher coherence, and black contours denote statistically significant regions (*P* < 0.05). The y-axis represents periodicity (years), and the x-axis represents calendar time.

Cutaneous leishmaniasis exhibited moderate and temporally heterogeneous coherence within the 4–6-year periodicity band, with significant coherence occurring intermittently throughout the study period (Fig. 2B).

Chagas disease showed weaker and more localized coherence within the 5–7-year periodicity band, with lower temporal persistence than that observed for malaria and cutaneous leishmaniasis (Fig. 2C).

Across diseases, significant coherence was concentrated within ENSO-relevant interannual periodicities, with disease-specific differences reflected in the dominant periodicity, magnitude, and temporal persistence of coherence patterns.

### 3.3 Structural temporal changes

Segmented regression identified multiple inflection points in disease trajectories, indicating changes in long-term incidence trends over the study period (Fig. 3). For malaria, inflection points were identified in 1983, 1988, 1997, 2000, and 2016. Cutaneous leishmaniasis exhibited inflection points in 1985, 1993, 2002, 2009, and 2016. Chagas disease showed inflection points in 2000, 2008, and 2015.

**Fig. 3:**
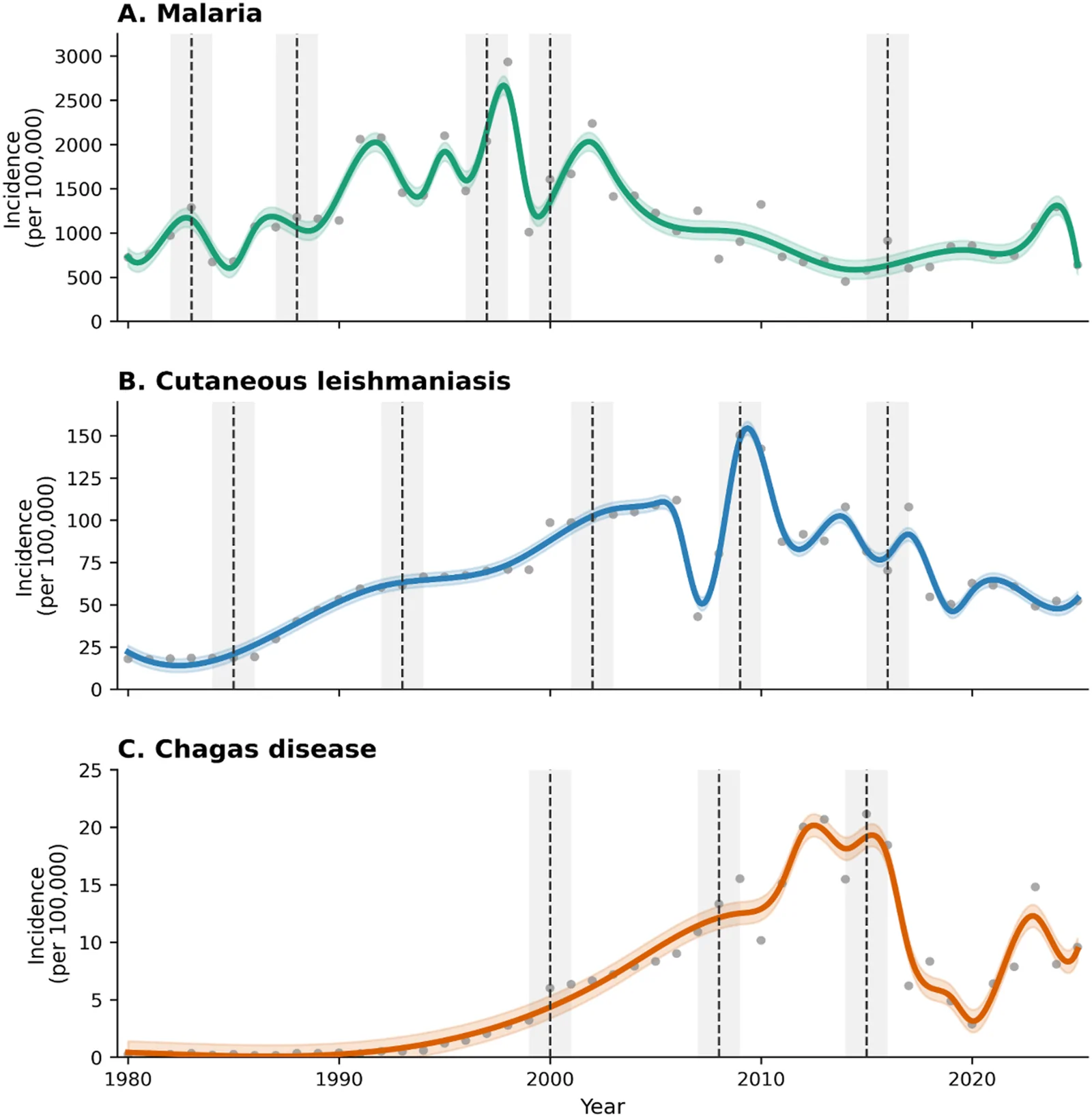
**Structural temporal changes in malaria, cutaneous leishmaniasis, and Chagas disease incidence in Colombia, 1980–2025.** Segmented regression models identifying temporal inflection points in annual incidence for (A) malaria (B) cutaneous leishmaniasis, and (C) Chagas disease. Points represent observed annual incidence, solid lines indicate fitted segmented trends, shaded areas represent 95% confidence intervals, and dashed vertical lines denote estimated inflection points. Corresponding estimates and bootstrap confidence intervals are reported in Supplementary Table S5.

These transitions were characterized by changes in slope magnitude, reflecting periods of acceleration or deceleration in disease incidence trajectories. Several inflection points occurred during or near intervals of significant wavelet coherence within interannual periodicities (Fig. 2). Although segmented regression and wavelet coherence analyses were performed independently, the temporal correspondence observed between several inflection points and intervals of significant ENSO–disease coherence suggests concordance between structural changes in long-term disease trajectories and periods of enhanced climatic synchronization**.** Detailed breakpoint estimates, including bootstrap CIs and associated slope changes (Δβ), are provided in Supplementary Table S5.

### 3.4 Spatial suitability and disease distribution

#### 3.4.1 Ecological suitability patterns

MaxEnt ecological niche models indicated heterogeneous environmental suitability across the six vector species analyzed (Fig. 4). *Anopheles albimanus*, *An. darlingi*, and *An. nuneztovari* s.l. exhibited high suitability in lowland regions, particularly in the Pacific, Amazon, and inter-Andean valleys, with complementary spatial distributions across these regions.

**Fig. 4:**
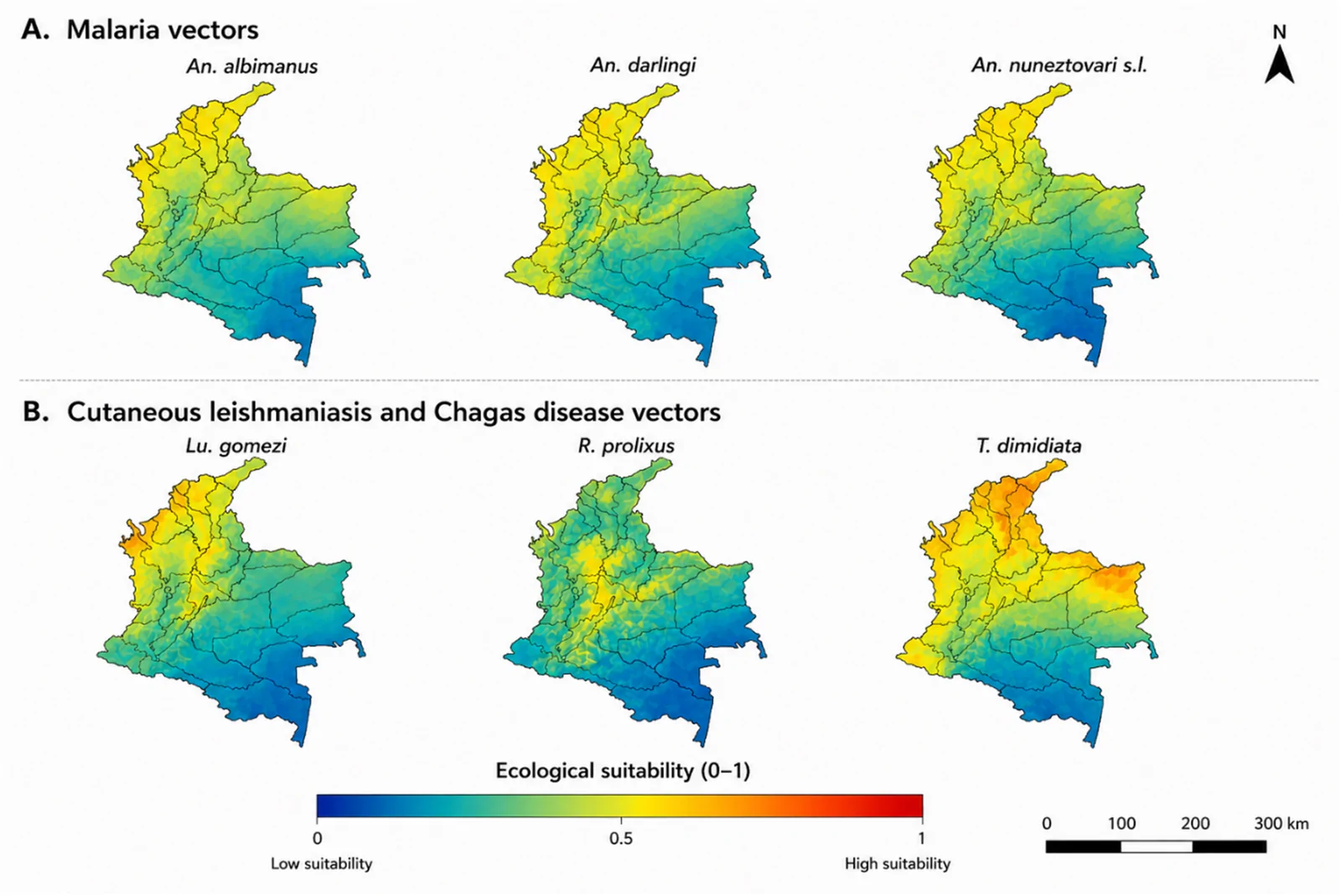
**Ecological suitability of *Anopheles albimanus*, *An. darlingi*, *An. nuneztovari* s.l., *Lutzomyia gomezi*, *Rhodnius prolixus*, and *Triatoma dimidiata* in Colombia, 1980–2025.** Species-specific ecological niche models are shown for (A) *An. albimanus*, *An. darlingi*, and *An. nuneztovari* s.l.; and (B) *Lu. gomezi*, *R. prolixus*, and *T. dimidiata*. Maps represent modeled environmental suitability on a continuous scale from 0 to 1, with higher values indicating greater environmental suitability.

*Lutzomyia gomezi*, *R. prolixus*, and *Triatoma dimidiata* exhibited more spatially structured suitability patterns. *Lu. gomezi* showed broad suitability across foothill and transitional ecosystems, whereas *R. prolixus* and *T. dimidiata* were concentrated primarily within inter-Andean and northern regions.

Partial spatial overlap among environmentally suitable areas for the six vector species was observed in several transitional ecological zones, particularly between lowland and foothill regions.

#### 3.4.2 Spatial distribution of disease incidence

Disease incidence exhibited distinct spatial clustering patterns across Colombia (Fig. 5). Malaria was concentrated primarily within Pacific and Amazon regions, whereas cutaneous leishmaniasis showed higher incidence in Andean foothill and forest-transition areas. Chagas disease displayed a broader and more diffuse spatial distribution, with higher incidence concentrated in historically endemic regions.

**Fig. 5:**
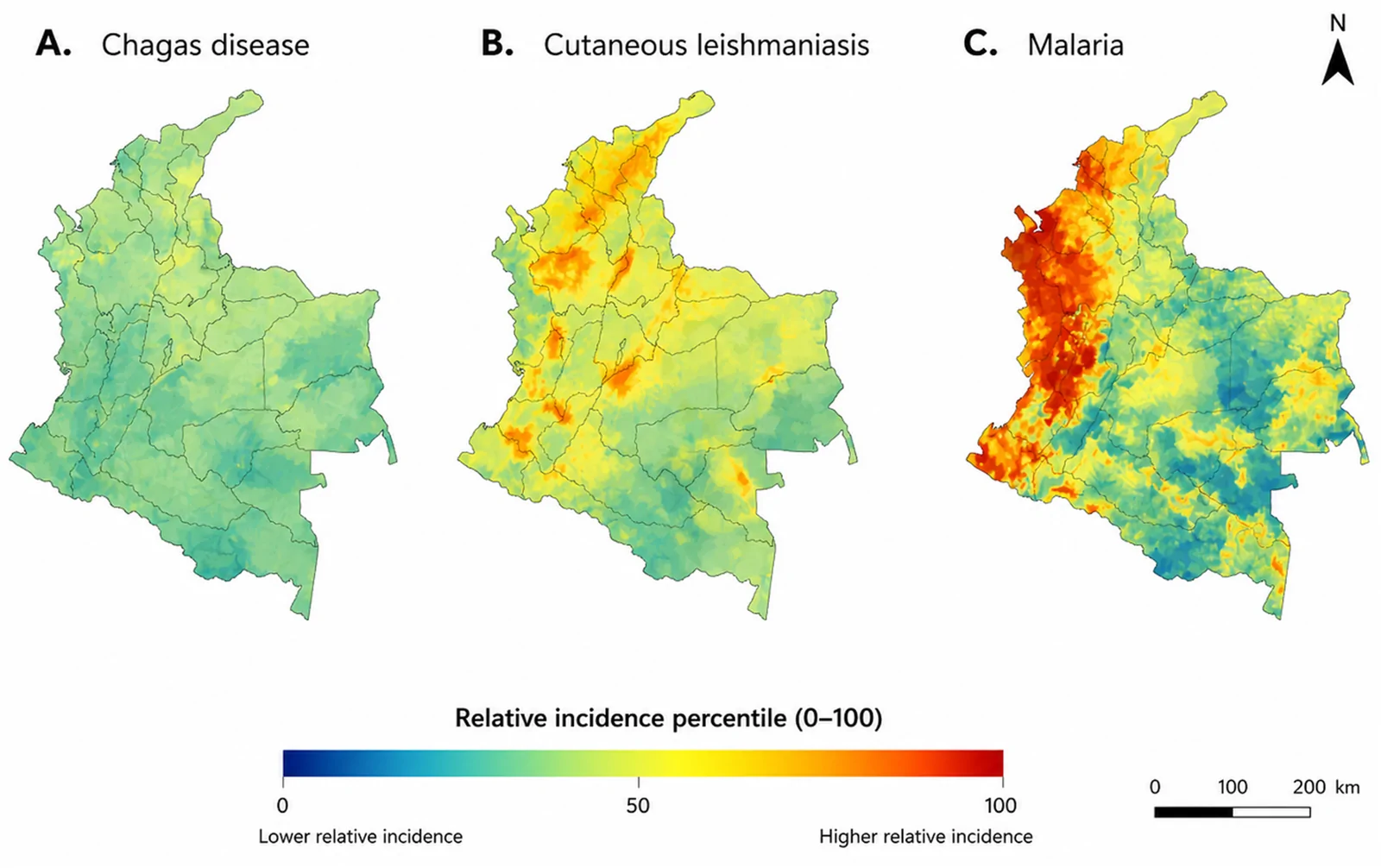
**Spatial distribution of relative incidence of Chagas disease, cutaneous leishmaniasis, and malaria in Colombia, 1980–2025.** Disease-specific incidence rates were transformed into percentile ranks (0–100) for (A) Chagas disease (B) cutaneous leishmaniasis, and (C) malaria. Percentiles were calculated independently for each disease, with higher values indicating higher relative incidence within the corresponding disease-specific distribution. Percentile values therefore represent relative spatial patterns and should not be interpreted as equivalent absolute incidence levels across diseases.

Spatial correspondence between observed disease incidence and modeled environmental suitability varied across disease systems. Concordance was greatest for malaria, intermediate for cutaneous leishmaniasis, and comparatively lower for Chagas disease.

Temperature-related variables were the dominant predictors of environmental suitability, with mean annual temperature and temperature seasonality together accounting for 64.3% of the mean relative predictor contribution across vector species models. Other environmental predictors contributed to a lesser extent (Supplementary Table S6).

### 3.5 Socioecological associations with disease incidence

Generalized linear mixed-effects models identified associations between disease incidence and climatic, environmental, and socioeconomic predictors across the disease systems analyzed (Supplementary Table S7). Temperature anomalies were positively associated with incidence (incidence rate ratio [IRR] = 1.07; 95% *CI*: 1.04–1.10), whereas precipitation anomalies were inversely associated (IRR = 0.96; 95% *CI*: 0.94–0.98). Deforestation rate (IRR = 1.06; 95% *CI*: 1.03–1.10) and poverty (IRR = 1.02; 95% *CI*: 1.00–1.04) were also positively associated with incidence, whereas health access showed an inverse association (IRR = 0.94; 95% *CI*: 0.91–0.98).

Interaction terms indicated heterogeneity in these associations across disease systems and local conditions. For malaria, the association with temperature was stronger in municipalities with deforestation levels at or above the national median (interaction IRR = 1.41; 95% *CI*: 1.18–1.68). For cutaneous leishmaniasis, poverty modified the association between precipitation variability and incidence (interaction IRR = 1.11; 95% *CI*: 1.04–1.18). Additional interactions between temperature and poverty and between precipitation and deforestation are reported in Supplementary Table S7.

### 3.6 Projected environmental suitability under future climate scenarios for 2035

Climate projections indicated heterogeneous changes in environmentally suitable areas across disease systems and emission scenarios. Between 2025 and 2035, high-suitability areas for malaria increased by 6.2%, 8.0%, and 9.8% under SSP1–2.6, SSP2–4.5, and SSP5–8.5, respectively. Corresponding increases for cutaneous leishmaniasis were 8.8%, 12.8%, and 16.8%. In contrast, high-suitability areas for Chagas disease decreased by 24.9%, 21.7%, and 14.7% across the same scenarios. Suitability-weighted mean elevations increased by 87.5, 103, and 119 m under SSP1–2.6, SSP2–4.5, and SSP5–8.5, respectively.

Despite these disease-specific differences, spatial convergence among projected suitability surfaces remained high. Mean pairwise Schoener's *D* was 0.886 under SSP1–2.6, SSP2–4.5, and SSP5–8.5. Disease-specific ensemble projections showed heterogeneous spatial responses across disease systems (Fig. 6), with the largest increases in high-suitability area observed for cutaneous leishmaniasis, followed by malaria.

**Fig. 6:**
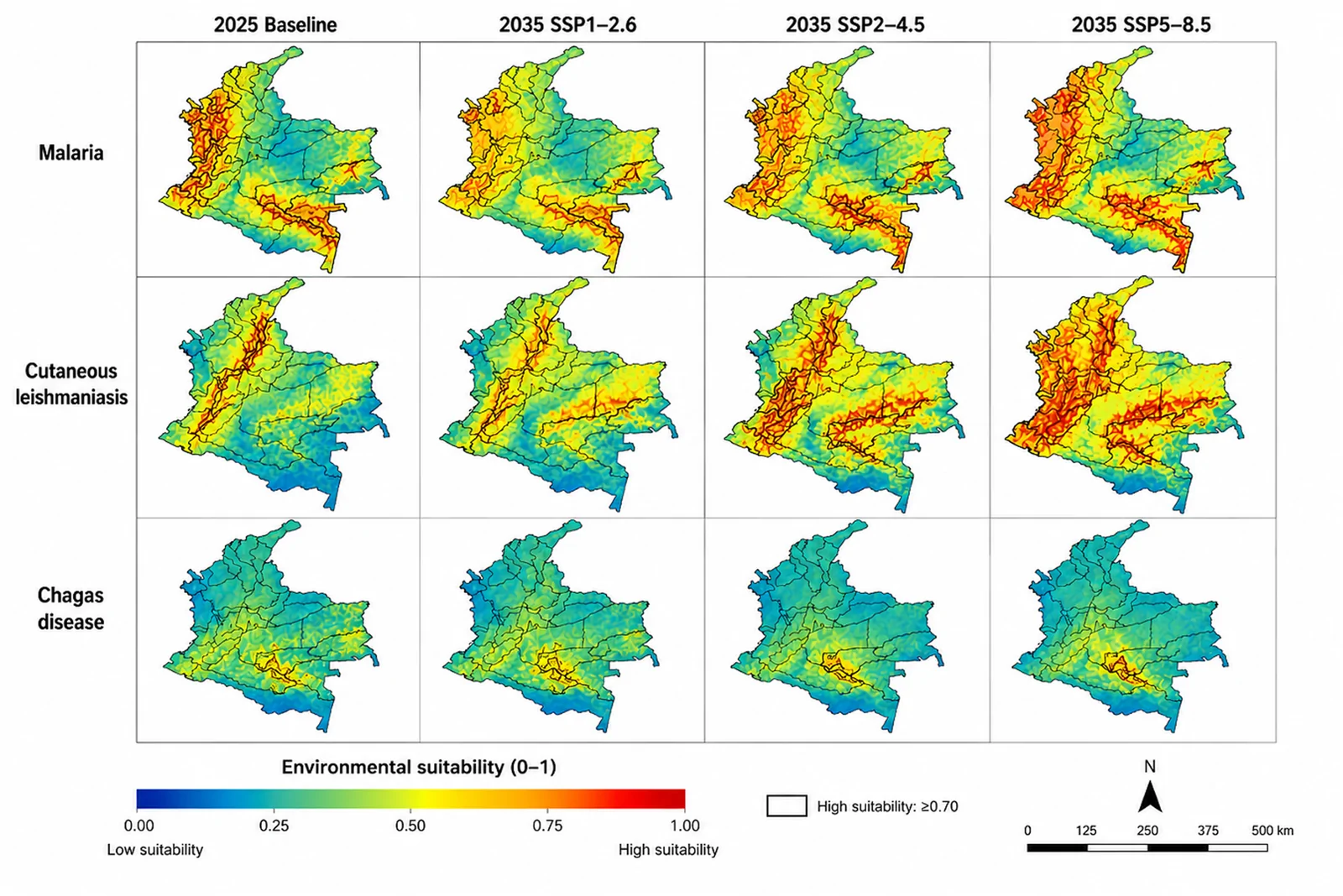
**Projected environmental suitability for malaria, cutaneous leishmaniasis, and Chagas disease in Colombia under future climate scenarios for 2035.** Disease-specific ensemble suitability surfaces are shown for the 2025 baseline and projections for 2035 under Shared Socioeconomic Pathway scenarios (SSP1–2.6, SSP2–4.5, and SSP5–8.5). Future climatic conditions were derived from the MPI-ESM1-2-HR global climate model. Suitability values range from 0 (low suitability) to 1 (high suitability), with high-suitability areas defined as cells with ensemble suitability values ≥ 0.70. Black contour lines delineate high-suitability areas.

Areas of multi-vector co-occurrence were estimated at 36,185, 43,922, and 52,142 km^2^ under SSP1–2.6, SSP2–4.5, and SSP5–8.5, respectively. The corresponding populations residing within these zones were approximately 1.67, 2.09, and 2.62 million, respectively.

Detailed projections of suitability changes, population exposure estimates, and multi-vector overlap metrics are presented in Supplementary Table S8. Species-specific vector suitability projections under SSP5–8.5 are presented in Supplementary Fig. S2.

### 3.7 Integrated climatic, temporal, and spatial indicators

Temporal synchronization indicators revealed a consistent hierarchy across diseases (Table 3). Malaria exhibited the highest mean wavelet coherence with ONI and the greatest number of significant coherence periods. Cutaneous leishmaniasis showed intermediate temporal coherence, whereas Chagas disease displayed weaker and less persistent coherence. Differences in dominant periodicity further indicated disease-specific temporal responses to ENSO variability.

**Table 3:** Temporal indicators of macroecological synchronization across vector-borne diseases in Colombia, 1980–2025.

Metric	Malaria	Cutaneous leishmaniasis	Chagas disease
Dominant periodicity (years)	3–5	4–6	5–7
Mean wavelet coherence (ONI vs. incidence)	0.62	0.48	0.31
Number of significant coherence periods	3	2	1

**Note:** Mean wavelet coherence summarizes temporal coupling between standardized annual ONI anomalies and disease incidence within the prespecified 2–8-year periodicity range.

**Abbreviations:** ENSO, El Niño–Southern Oscillation; ONI, Oceanic Niño Index.

Spatial overlap among disease-specific suitability surfaces remained high under future climate scenarios, with mean pairwise Schoener's *D* = 0.886 under SSP1–2.6, SSP2–4.5, and SSP5–8.5, indicating substantial spatial similarity among projected suitability surfaces despite heterogeneous disease-specific changes in the extent of high-suitability areas.

### 3.8 Model validation and uncertainty

Negative binomial sensitivity analyses of the monthly DLNMs yielded estimates broadly consistent with the primary quasi-Poisson models, particularly for the delayed malaria–temperature association (Supplementary Table S4). Ecological niche models showed high discriminatory performance, with AUC values ranging from 0.86 to 0.94 and TSS values ranging from 0.70 to 0.84 across vector species models (Supplementary Table S6).

## 4 Discussion

This study presents an integrated framework for understanding how large-scale climatic variability structures the dynamics of multiple vector-borne parasitic diseases across Colombia. By integrating long-term epidemiological, climatic, and ecological datasets, the analyses indicate that ENSO-related climatic variability is associated with temporal synchronization and geographic redistribution of environmental suitability across malaria, cutaneous leishmaniasis, and Chagas disease over four decades. ENSO-related variability was associated with disease dynamics to differing degrees across diseases, with a one-unit increase in ONI corresponding to a 0.38 °C rise in temperature, a 42 mm decrease in precipitation, and increases of 6.1% and 4.3% in malaria and cutaneous leishmaniasis incidence, respectively. Models incorporating local climatic anomalies explained 57.3% of the deviance in malaria incidence, 55.7% in cutaneous leishmaniasis, and 45.6% in Chagas disease, consistent with heterogeneous climatic sensitivity across transmission systems. A gradient in climatic sensitivity emerges across diseases, with malaria exhibiting the strongest temporal coupling, cutaneous leishmaniasis intermediate sensitivity, and Chagas disease weaker climatic associations and lower temporal coherence with ENSO. Monthly distributed lag analyses further identified a robust delayed temperature association for malaria, whereas delayed associations were less consistent for cutaneous leishmaniasis and were not statistically significant for Chagas disease under autocorrelation-robust inference. These findings are consistent with ENSO acting as a macroecological forcing mechanism associated with heterogeneous responses across transmission systems and spatial and temporal scales under changing environmental conditions.

Previous national studies have characterized the long-term spatiotemporal epidemiology of cutaneous, mucosal, and visceral leishmaniasis in Colombia [17] and examined associations between El Niño, climatic conditions, deforestation, incidence, and outbreak occurrence across multiple vector-borne diseases [10]. The present analysis extends this evidence by examining malaria, cutaneous leishmaniasis, and Chagas disease within a common macroecological framework that integrates interannual ENSO variability, delayed climatic associations, temporal coherence, vector ecological suitability, municipality-level socioecological conditions, and future climate projections. This broader comparative approach allows disease-specific responses to climatic variability to be evaluated alongside patterns of temporal and spatial convergence across parasitic transmission systems.

At a conceptual level, the relationship between large-scale climatic variability and disease incidence follows a hierarchical framework in which macroclimatic forcing influences transmission indirectly through environmental modulation rather than direct epidemiological triggering. Persistent wavelet coherence was observed within ENSO-relevant interannual periodicities, with differences in dominant periodicity and coherence strength among disease systems. Several segmented regression inflection points also temporally corresponded with intervals of enhanced climatic synchronization, suggesting that some structural changes in disease trajectories occurred during periods of stronger climate–disease coupling. These analyses do not establish causality, and the observed temporal coherence between ENSO variability and disease incidence should not be interpreted as direct causation linking climatic oscillations to individual outbreak events. Instead, ENSO operates as a large-scale environmental forcing that shapes background conditions across regions, with the observed synchrony reflecting parallel responses of independent transmission systems to common environmental variability rather than simultaneous epidemiological causation.

ENSO phases modulate temperature, humidity, and hydrological patterns that influence vector ecology [[31], [32], [33]]. During warm events, reduced rainfall and elevated temperatures increase the persistence of residual water bodies, conditions that favor *Anopheles* larval development and contribute to synchronized malaria resurgence [[34], [35], [36]]. Temperature also affects mosquito development, aging, and other biological processes relevant to vectorial transmission, although these responses are species- and temperature-dependent [[37], [38], [39]]. These mechanisms provide a plausible explanation for the observed synchronization across disease systems, linking climatic anomalies with coordinated epidemiological responses rather than isolated local outbreaks. Consistent with these mechanisms, temperature showed the strongest positive climatic association (IRR = 1.07), whereas precipitation showed an inverse association (IRR = 0.96), highlighting the importance of hydroclimatic balance in shaping vector habitat suitability.

The observed gradient in climatic sensitivity reflects fundamental ecological differences among transmission systems. Malaria vectors, characterized by high mobility and rapid life cycles, respond more directly to short-term climatic variability, whereas cutaneous leishmaniasis vectors exhibit intermediate sensitivity due to combined dependence on climatic conditions and habitat structure. In contrast, Chagas disease transmission is structured by longer–term processes, including domestic colonization dynamics and reservoir ecology, resulting in weaker climatic associations. This differentiation was also reflected in the monthly distributed lag analyses: temperature anomalies were associated with increased malaria incidence over lags 3–8 months, whereas associations were less consistent for cutaneous leishmaniasis and no statistically significant delayed associations were identified for Chagas disease under HAC inference. These differences are consistent with reduced temporal coherence and lower deviance explained for Chagas disease, as well as the absence of a statistically significant ONI–incidence association for this disease. Non-stationary wavelet coherence patterns further indicate that climate–disease relationships vary over time and ecological context, reflecting the dynamic nature of coupled environmental and epidemiological systems.

Chagas disease provides a useful example of the interaction between climatic variability, vector control, and ecological adaptation. Following the certified elimination of *R. prolixus* from domiciliary transmission in most endemic areas of Colombia [40], *T. dimidiata* has assumed increasing epidemiological importance in areas where domiciliary transmission by *R. prolixus* has been interrupted [21,41,42]. Unlike malaria and cutaneous leishmaniasis, whose vectors may respond more rapidly to climatic variability, Chagas disease transmission is structured by slower ecological and social processes, which may contribute to its weaker and less consistent climatic associations across temporal scales [43,44]. The absence of statistically significant delayed associations in the monthly DLNMs is consistent with a transmission system in which short-term climatic variability may be less influential than vector colonization, reservoir dynamics, housing conditions, and sustained vector-control interventions. These findings suggest that sustained vector-control interventions and changes in the relative epidemiological importance of vector species may modify the climatic responsiveness of Chagas disease transmission, contributing to the weaker short-term climatic associations observed in this study.

The magnitude of disease responses to climatic variability is further modulated by socioeconomic and environmental context [19]. Deforestation and socioeconomic vulnerability may shape local transmission conditions [10,45,46], while landscape transformation can modify local environmental conditions, alter vector habitats, and influence patterns of human exposure [[47], [48], [49]]. These mechanisms are consistent with model outputs in which deforestation (IRR = 1.06) and poverty (IRR = 1.02) were positively associated with disease incidence. Higher health access was inversely associated with reported disease incidence (IRR = 0.94), although this ecological association should be interpreted cautiously because differences in healthcare accessibility, surveillance, and case detection may influence reported incidence across municipalities. For malaria, recent evidence from Colombia further indicates that residual *P. falciparum* transmission is spatially concentrated within persistent territorial systems, particularly in the Pacific region [50]. Together, these findings indicate that climatic forcing operates within broader socioecological systems, where environmental transformation and social vulnerability may amplify or attenuate climatic associations with disease transmission across heterogeneous landscapes [4,5,51].

Spatial analyses further indicate that climatic variability may influence not only temporal disease dynamics but also the long-term geographic organization of transmission risk. Independent ecological niche models projected heterogeneous changes in environmentally suitable areas across disease systems under future climate scenarios. High-suitability areas increased for malaria and cutaneous leishmaniasis, with progressively larger expansions under higher-emission scenarios, whereas Chagas disease showed a contraction across all three scenarios. Despite these contrasting disease-specific trajectories, projected suitability surfaces retained substantial spatial overlap, and the extent of multi-vector co-occurrence was greater under higher-emission scenarios. These patterns suggest that future climatic change may reorganize transmission potential and increase opportunities for spatial convergence among vector systems without requiring parallel expansion of suitable areas for all diseases. Rather than representing direct predictions of future disease burden, these projections identify areas where environmental suitability may increase if ecological and epidemiological conditions also permit sustained transmission. The projected increase in suitability-weighted mean elevation further suggests a redistribution of environmentally favorable conditions along altitudinal gradients. This pattern is consistent with previous evidence from Colombia showing altitudinal changes in malaria distribution associated with warmer conditions and projected altitudinal shifts in leishmaniasis vector suitability under future climate scenarios [52,53].

Environmental suitability should not be interpreted as equivalent to observed disease transmission. Environmentally favorable conditions may not result in reported human cases because transmission also depends on vector presence and abundance, reservoir dynamics, human exposure, surveillance and diagnostic capacity, and other biological and social determinants [53,54]. This distinction highlights the value of integrating ecological suitability models with epidemiological surveillance, vector monitoring, and field validation to identify areas where changing environmental conditions may warrant strengthened surveillance and targeted public health interventions [55,56]. Accordingly, future climate projections should be interpreted as estimates of changing environmental suitability rather than deterministic forecasts of disease incidence, as transmission will continue to depend on interactions among climatic conditions, vector ecology, environmental transformation, and socioeconomic factors.

Several limitations should be considered. First, the use of long-term surveillance data may be affected by temporal and geographic variation in diagnostic practices, reporting intensity, and case ascertainment, particularly for Chagas disease. Second, gridded climatic and environmental data and municipality-level socioeconomic indicators may not capture fine-scale heterogeneity in exposure and vulnerability. Third, residual temporal autocorrelation remained detectable in the monthly distributed lag models despite adjustment for seasonality and long-term trends; HAC inference and negative binomial sensitivity analyses were therefore used to assess robustness. Fourth, ecological niche models estimate potential environmental suitability rather than realized transmission and may be influenced by spatial variation in vector sampling. Finally, although three CMIP6 scenarios (SSP1–2.6, SSP2–4.5, and SSP5–8.5) were evaluated, projections were based on a single global climate model (MPI-ESM1-2-HR) and therefore do not capture the full range of inter-model uncertainty. Accordingly, projected suitability should be interpreted as potential environmental change rather than as a direct forecast of future disease incidence.

## 5 Conclusion

Climatic variability, environmental transformation, and social vulnerability are jointly associated with the dynamics of malaria, cutaneous leishmaniasis, and Chagas disease in Colombia. Across complementary analytical approaches, the findings are consistent with ENSO acting as a macroecological forcing mechanism associated with temporal synchronization and spatially heterogeneous responses across transmission systems.

A gradient in climatic sensitivity, strongest for malaria, intermediate for cutaneous leishmaniasis, and weaker for Chagas disease, reflects fundamental differences in vector ecology and transmission dynamics and is consistent with a hierarchical structure linking large-scale climatic variability to local environmental conditions.

The integration of temporal, climatic, ecological, and spatial evidence provides a comparative framework for understanding environmentally mediated transmission dynamics across multiple vector-borne parasitic diseases. Under future climate scenarios, projected changes in environmental suitability and the spatial convergence of suitable areas underscore the value of anticipatory, climate-informed surveillance strategies capable of addressing geographically overlapping disease risks.

## CRediT authorship contribution statement

**Mario J. Olivera:** Writing – review & editing, Writing – original draft, Supervision, Methodology, Investigation, Formal analysis, Data curation, Conceptualization. **Julián Felipe Porras-Villamil:** Writing – review & editing, Methodology, Investigation, Formal analysis, Data curation. **Màrius Vicent Fuentes:** Writing – review & editing, Writing – original draft, Methodology, Investigation, Funding acquisition, Conceptualization.

## Availability of data and materials

The results presented are sufficient to support the conclusions of this study. Nonetheless, the lead author is available to provide additional data upon request.

## Funding

This work was supported by the Instituto Nacional de Salud, Colombia.

## Declaration of competing interest

The authors declare that they have no known competing financial interests or personal relationships that could have appeared to influence the work reported in this paper.

## Figures and Tables

**Figure:**
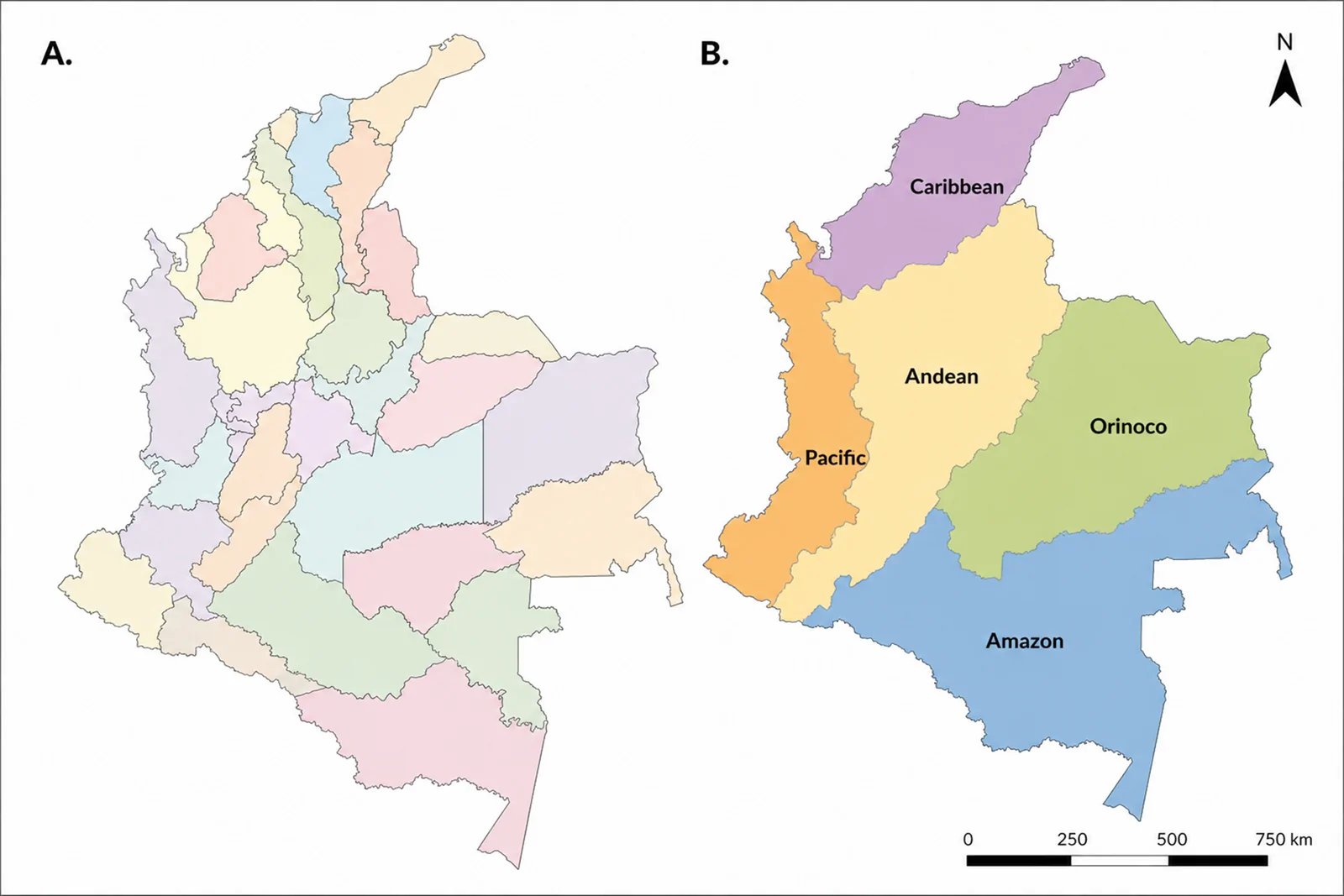
figs1

**Figure:**
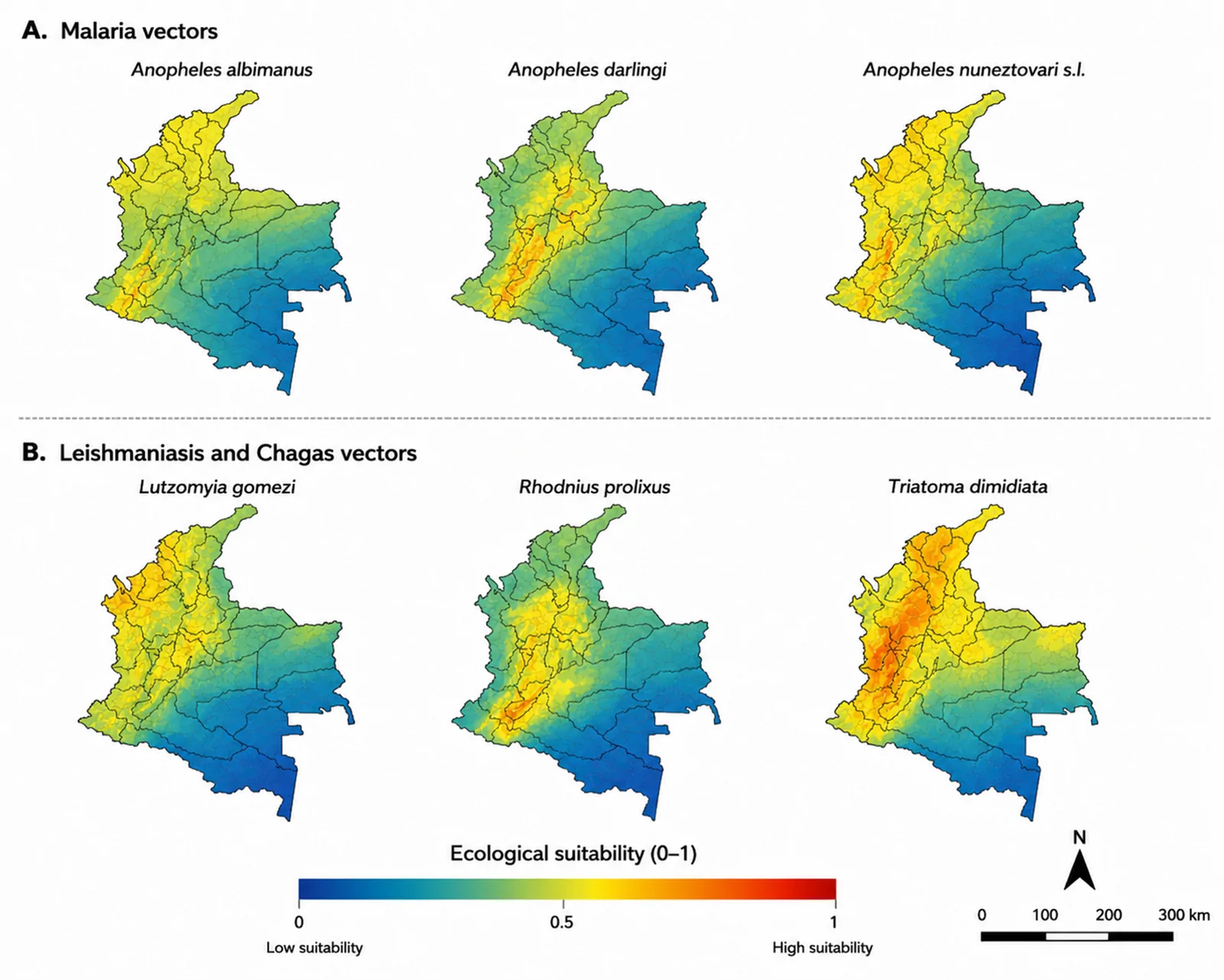
figs2
