# Supplementary material for: Macroecological synchronization of vector-borne diseases under climate variability in Colombia
Source: Sci One Health. 2026 Sep 5;5:100169. doi: 10.1016/j.soh.2026.100169 (PMC13652968; doi:10.1016/j.soh.2026.100169)
Supplement: Supplementary file 1 [file mmc2.docx]

**Supplementary Table S1. Climatic, environmental, and socioeconomic predictors included in the analytical framework.**

| **Category** | **Predictor** | **Code** | **Units** | **Spatial/temporal resolution** | **Analytical role** |
| --- | --- | --- | --- | --- | --- |
| Climate | Mean annual temperature | BIO1 | °C | 1 km² | Thermal suitability in ecological niche models |
| Climate | Temperature seasonality | BIO4 | SD (°C) | 1 km² | Seasonal climatic constraint on vector suitability |
| Climate | Precipitation of wettest quarter | BIO16 | mm | 1 km² | Hydrological conditions relevant to vector habitat suitability |
| Climate | Temperature anomaly | — | °C anomaly | Monthly; annually aggregated where required | Monthly DLNM and annual temporal analyses |
| Climate | Precipitation anomaly | — | mm anomaly | Monthly; annually aggregated where required | Monthly DLNM and annual temporal analyses |
| Climate | Relative humidity | RH | % | 1 km² | Environmental predictor in ecological niche models |
| Climate | Relative humidity anomaly | — | Percentage-point anomaly | Monthly; annually aggregated where required | Monthly DLNM and annual temporal analyses |
| Climate | Oceanic Niño Index | ONI | °C anomaly | Monthly; annually aggregated for annual analyses | ENSO forcing and interannual climatic variability |
| Landscape | Normalized Difference Vegetation Index | NDVI | Index (−1 to 1) | 1 km² | Vegetation structure and ecological suitability |
| Landscape | Annual deforestation rate | DEFOR | % yr⁻¹ | 1 km² / municipality | Habitat disturbance and socioecological modeling |
| Landscape | Elevation | ELEV | m a.s.l. | 1 km² | Topographic constraint on vector suitability |
| Socioeconomic | Poverty index | POV | % / index | Municipality | Socioeconomic vulnerability and municipality-level modeling |
| Socioeconomic | Health access index | HEALTH | % coverage / index | Municipality | Healthcare accessibility and municipality-level modeling |

**Note:** Monthly temperature, precipitation, and relative humidity anomalies were retained at monthly resolution for distributed lag analyses and aggregated by calendar year for annual temporal analyses. Monthly ONI values were aggregated for analyses of interannual ENSO variability. Municipality-level socioecological models used annual observations.

**Abbreviations:** DLNM, distributed lag nonlinear model; ENSO, El Niño–Southern Oscillation; NDVI, Normalized Difference Vegetation Index; ONI, Oceanic Niño Index; RH, relative humidity.
