# Supplementary material for: Macroecological synchronization of vector-borne diseases under climate variability in Colombia
Source: Sci One Health. 2026 Sep 5;5:100169. doi: 10.1016/j.soh.2026.100169 (PMC13652968; doi:10.1016/j.soh.2026.100169)
Supplement: Supplementary file 2 [file mmc3.docx]

**Supplementary Table S2. Long-term descriptive epidemiological characteristics of malaria, cutaneous leishmaniasis, and Chagas disease in Colombia, 1980–2025.**

| **Disease** | **Total cases (×10³)** | **Mean annual incidence (per 100,000)** | **Coefficient of variation (%)** |
| --- | --- | --- | --- |
| Malaria | 4,739.0 | 960.2 | 68.4 |
| Cutaneous leishmaniasis | 313.1 | 73.2 | 61.2 |
| Chagas disease | 16.1 | 4.7 | 78.5 |

**Note:** Total cases represent cumulative national reports over the study period derived from surveillance records of the National Institute of Health and the national public health surveillance system. Mean annual incidence summarizes the average incidence over the study period, whereas coefficients of variation characterize long-term temporal variability. Changes in surveillance coverage and diagnostic capacity over time, particularly for Chagas disease after 2000, should be considered when interpreting these estimates.
