# Supplementary material for: Macroecological synchronization of vector-borne diseases under climate variability in Colombia
Source: Sci One Health. 2026 Sep 5;5:100169. doi: 10.1016/j.soh.2026.100169 (PMC13652968; doi:10.1016/j.soh.2026.100169)
Supplement: Supplementary file 3 [file mmc4.docx]

**Supplementary Table S3. Associations between annual local climatic anomalies and disease incidence estimated using generalized additive models (Model 1).**

| **Disease** | **Temperature anomaly (% change per +1 °C)** | **95% CI** | **p-value** | **Precipitation anomaly (% change per +10 mm)** | **95% CI** | **p-value** | **Relative humidity anomaly (% change per +1 percentage point)** | **95% CI** | | **p-value** | **Deviance explained (%)** | |
| --- | --- | --- | --- | --- | --- | --- | --- | --- | --- | --- | --- | --- |
| Malaria | 4.8% | 1.7 – 8.1 | 0.005 | −3.2% | −5.6 – −0.8 | 0.014 | −0.9% | −2.0 – 0.1 | 0.095 | | 57.3 |  |
| Cutaneous leishmaniasis | 3.7% | 1.2 – 6.3 | 0.006 | −2.4% | −4.4 – −0.5 | 0.020 | −0.6% | −1.5 – 0.2 | 0.158 | | 55.7 |  |
| Chagas disease | −1.1% | −3.5 – 1.4 | 0.397 | −0.8% | −2.8 – 1.2 | 0.443 | −0.3% | −1.1 – 0.6 | 0.522 | | 45.6 |  |

**Note:** Estimates represent percentage changes in annual disease incidence associated with a +1 °C temperature anomaly, +10 mm precipitation anomaly, and +1 percentage-point relative humidity anomaly. Estimates were obtained from generalized additive models (Model 1) controlling for long-term temporal trends using a penalized smooth function of calendar year. Model 1 included local climatic anomalies without ONI.

**Abbreviations:** CI, confidence interval; ONI, Oceanic Niño Index.
