# Supplementary material for: Macroecological synchronization of vector-borne diseases under climate variability in Colombia
Source: Sci One Health. 2026 Sep 5;5:100169. doi: 10.1016/j.soh.2026.100169 (PMC13652968; doi:10.1016/j.soh.2026.100169)
Supplement: Supplementary file 4 [file mmc5.docx]

**Supplementary Table S4. Selected cumulative associations from distributed lag nonlinear models of monthly climatic anomalies and disease incidence in Colombia, 1980–2025.**

| **Disease** | **Climatic exposure** | **Contrast** | **RR, 0–12 months (HAC 95% CI)** | **RR, 3–8 months (HAC 95% CI)** | **NB sensitivity, 0–12 months (95% CI)** | **NB sensitivity, 3–8 months (95% CI)** |
| --- | --- | --- | --- | --- | --- | --- |
| Malaria | Temperature | +1 °C | 1.42 (0.92–2.18) | 1.28 (1.03–1.60) | 1.45 (1.08–1.97) | 1.30 (1.14–1.49) |
| Cutaneous leishmaniasis | Relative humidity | +1 pp | 0.81 (0.63–1.03) | 0.89 (0.78–1.00) | 0.78 (0.64–0.96) | 0.87 (0.79–0.96) |
| Chagas disease | Temperature | +1 °C | 0.29 (0.07–1.19) | 0.74 (0.37–1.49) | 0.34 (0.18–0.66) | 0.76 (0.57–1.01) |

**Note:** Relative risks were estimated using single-exposure quasi-Poisson distributed lag nonlinear models with a maximum lag of 12 months. Primary 95% confidence intervals were calculated using Newey–West heteroskedasticity- and autocorrelation-consistent covariance estimators with a 12-month lag. Negative binomial models with the same specification were fitted as sensitivity analyses. Estimates for 0–12 months represent cumulative associations across the complete prespecified lag horizon, whereas estimates for 3–8 months summarize delayed associations within this interval. RRs are reported according to the exposure contrasts indicated in the table.

**Abbreviations:** CI, confidence interval; DLNM, distributed lag nonlinear model; HAC, heteroskedasticity- and autocorrelation-consistent; NB, negative binomial; RR, relative risk.
