# Supplementary material for: Macroecological synchronization of vector-borne diseases under climate variability in Colombia
Source: Sci One Health. 2026 Sep 5;5:100169. doi: 10.1016/j.soh.2026.100169 (PMC13652968; doi:10.1016/j.soh.2026.100169)
Supplement: Supplementary file 5 [file mmc6.docx]

**Supplementary Table S5.** **Estimated temporal inflection points in national incidence trends of malaria, cutaneous leishmaniasis, and Chagas disease in Colombia, 1980–2025**

| **Disease** | **Inflection year** | **95% CI (bootstrap)** | **Change in slope (Δβ, log-incidence scale)** |
| --- | --- | --- | --- |
| Malaria | 1983 | 1982–1984 | 0.06 |
|  | 1988 | 1987–1989 | −0.08 |
|  | 1997 | 1996–1998 | 0.06 |
|  | 2000 | 1999–2001 | −0.13 |
|  | 2016 | 2015–2017 | −0.04 |
| Cutaneous leishmaniasis | 1985 | 1984–1986 | 0.07 |
|  | 1993 | 1992–1994 | 0.07 |
|  | 2002 | 2001–2003 | 0.07 |
|  | 2009 | 2008–2010 | −0.07 |
|  | 2016 | 2015–2017 | −0.06 |
| Chagas disease | 2000 | 1999–2001 | 0.14 |
|  | 2008 | 2007–2009 | −0.05 |
|  | 2015 | 2014–2016 | −0.05 |

**Note**: Inflection points were estimated using segmented regression, with 95% confidence intervals obtained by nonparametric bootstrap resampling (1,000 iterations). Changes in slope (Δβ) represent estimated differences in the log-incidence trend before and after each inflection point.

**Abbreviations:** CI, confidence interval.
