# Supplementary material for: Macroecological synchronization of vector-borne diseases under climate variability in Colombia
Source: Sci One Health. 2026 Sep 5;5:100169. doi: 10.1016/j.soh.2026.100169 (PMC13652968; doi:10.1016/j.soh.2026.100169)
Supplement: Supplementary file 6 [file mmc7.docx]

**Supplementary Table S6. Main environmental predictors of vector habitat suitability across ecological niche models in Colombia, 1980–2025.**

| **Predictor** | **Code** | **Mean contribution (%)** | **General response** | **Vector species with strongest response** |
| --- | --- | --- | --- | --- |
| Mean annual temperature | BIO1 | 34.5 | Positive, nonlinear | *An. albimanus*, *Lu. gomezi* |
| Temperature seasonality | BIO4 | 29.8 | Predominantly negative | *R. prolixus*, *T. dimidiata* |
| Precipitation of wettest quarter | BIO16 | 11.2 | Positive, nonlinear | *An. darlingi* |
| Normalized Difference Vegetation Index | NDVI | 8.7 | Positive at intermediate values | *Lu. gomezi* |
| Annual deforestation rate | DEFOR | 7.9 | Positive | *An. nuneztovari* s.l*.* |
| Elevation | ELEV | 4.5 | Predominantly negative | All *Anopheles* spp. |

**Note:** Predictor contributions were derived from ecological niche models fitted using MaxEnt-based algorithms and represent the mean relative contribution of the main predictors reported across vector species models. Model performance ranged from AUC = 0.86–0.94 and TSS = 0.70–0.84. BIO variables correspond to WorldClim bioclimatic predictors.

**Abbreviations:** AUC, area under the receiver operating characteristic curve; BIO1, mean annual temperature; BIO4, temperature seasonality; BIO16, precipitation of the wettest quarter; DEFOR, annual deforestation rate; ELEV, elevation; MaxEnt, maximum entropy; NDVI, Normalized Difference Vegetation Index; TSS, True Skill Statistic.
