# Supplementary material for: Macroecological synchronization of vector-borne diseases under climate variability in Colombia
Source: Sci One Health. 2026 Sep 5;5:100169. doi: 10.1016/j.soh.2026.100169 (PMC13652968; doi:10.1016/j.soh.2026.100169)
Supplement: Supplementary file 7 [file mmc8.docx]

**Supplementary Table S7. Municipality-level generalized linear mixed-effects model estimates of climatic, environmental, and socioeconomic associations with vector-borne disease incidence in Colombia, 1980–2025.**

| **Term** | **Disease system** | **Scaling** | **β (log)** | **IRR** | **95% CI (IRR)** | **p-value** |
| --- | --- | --- | --- | --- | --- | --- |
| **Main effects** |  |  |  |  |  |  |
| Temperature anomaly | All systems | per 1 °C | 0.064 | 1.07 | 1.04–1.10 | <0.001 |
| Precipitation anomaly | All systems | per 10 mm | −0.043 | 0.96 | 0.94–0.98 | 0.002 |
| Deforestation rate | All systems | per 1 % annual | 0.058 | 1.06 | 1.03–1.10 | <0.001 |
| Poverty index | All systems | per 10 index units | 0.021 | 1.02 | 1.00–1.04 | 0.046 |
| Health access index | All systems | per 10 index units | −0.060 | 0.94 | 0.91–0.98 | 0.041 |
| **Interaction effects** |  |  |  |  |  |  |
| Temperature anomaly × high deforestation | Malaria | per 1 °C in municipalities ≥ national median deforestation | 0.344 | 1.41 | 1.18–1.68 | <0.001 |
| Poverty index × precipitation anomaly | Cutaneous leishmaniasis | per 10 poverty units × 10 mm rainfall anomaly | 0.104 | 1.11 | 1.04–1.18 | 0.001 |
| Temperature anomaly × poverty index | All systems | per 1 °C × 10 poverty units | 0.052 | 1.05 | 1.01–1.11 | 0.028 |
| Precipitation anomaly × deforestation rate | Malaria / Leishmaniasis | per 10 mm × 1 % deforestation | 0.037 | 1.04 | 1.01–1.08 | 0.041 |

**Note:** Estimates were obtained from municipality-level generalized linear mixed-effects models with a negative binomial distribution using annual observations. Annual disease counts were modeled with the disease-specific population at risk included as a log offset and municipality included as a random intercept. IRRs represent exponentiated regression coefficients [exp(β)] according to the scaling indicated for each term. Interaction terms represent departures from the corresponding main effects on the multiplicative incidence-rate scale.

**Abbreviations:** CI, confidence interval; GLMM, generalized linear mixed-effects model; IRR, incidence rate ratio.
