# Supplementary material for: Macroecological synchronization of vector-borne diseases under climate variability in Colombia
Source: Sci One Health. 2026 Sep 5;5:100169. doi: 10.1016/j.soh.2026.100169 (PMC13652968; doi:10.1016/j.soh.2026.100169)
Supplement: Supplementary file 8 [file mmc9.docx]

**Supplementary Table S8. Projected changes in disease-specific environmental suitability and multi-vector spatial co-occurrence under CMIP6 climate scenarios in Colombia, 2035.**

| **Scenario** | **Malaria high-suitability area (%)** | **Cutaneous leishmaniasis high-suitability area (%)** | **Chagas disease high-suitability area (%)** | **Mean centroid elevation change (m)** | **Mean Schoener’s D** | **Multi-vector co-occurrence area (km²)** | **Population residing in multi-vector suitability zones (×10⁶)** |
| --- | --- | --- | --- | --- | --- | --- | --- |
| SSP1–2.6 | 6.2 | 8.8 | −24.9 | 87.5 | 0.886 | 36,185 | 1.67 |
| SSP2–4.5 | 8.0 | 12.8 | −21.7 | 103 | 0.886 | 43,922 | 2.09 |
| SSP5–8.5 | 9.8 | 16.8 | −14.7 | 119 | 0.886 | 52,142 | 2.62 |

**Note:** Projections were derived from ecological niche models forced with CMIP6 climate projections from the MPI-ESM1-2-HR global climate model. Changes in high-suitability area represent percentage differences between 2025 and 2035 in areas with suitability ≥0.70 for each disease-specific suitability surface. Centroid elevation change represents the mean scenario-specific change in suitability-weighted elevation across disease systems. Schoener’s D represents the mean of the three pairwise comparisons among normalized continuous disease-specific suitability surfaces. Multi-vector co-occurrence area and population estimates represent areas where environmental suitability for multiple vector systems overlaps. Non-climatic predictors were held constant across future projections. Values represent changes in environmental suitability and should not be interpreted as forecasts of disease incidence.

**Abbreviations:** CMIP6, Coupled Model Intercomparison Project Phase 6; SSP, Shared Socioeconomic Pathway
